# Metagenomic data from gutter water in the city of Pointe-Noire, Republic of Congo

**DOI:** 10.1016/j.dib.2024.110655

**Published:** 2024-06-27

**Authors:** Bouziane Moumen, Céline Samba-Louaka, Victoire Aubierge Matondo Kimpamboudi, Anicet Magloire Boumba, Hervé Sabin Ngoma, Ascel Samba-Louaka

**Affiliations:** a Université de Poitiers, UMR CNRS 7267, Laboratoire Ecologie et Biologie des Interactions, Poitiers, France; b Centre Médical CMC Medico, Pointe-Noire, Congo; c Direction Départementale des Soins et Services de Santé de Pointe Noire, Pointe-Noire, Congo

**Keywords:** Antibiotic-resistance genes, Shotgun metagenomics, Gutter water, Central Africa

## Abstract

After Amazonia, the Congo Basin represents the second-largest tropical rainforest area in the world. This basin harbours remarkable biodiversity, yet much of its microbiological diversity within its waters, soils, and populations remains largely unexplored and undiscovered. While many initiatives to characterize global biodiversity are being undertaken, few are conducted in Africa and none of them concern the Congo Basin specifically in urban areas. In this context, we assessed the microbial diversity present in gutter water in the city of Pointe-Noire, Congo. This town has interesting characteristics as the population density is high and it is located between the Atlantic Ocean and the forest of Mayombe in Central Africa. The findings illuminate the microbial composition of surface water in Pointe-Noire. The dataset allows the identification of putative new bacteria through the assembly of 81 meta-genome-assembled genomes. It also serves as a valuable primary resource for assessing the presence of antibiotic-resistant genes, offering a useful tool for monitoring risks by public health authorities.

Specifications Table

**Table:** 

Subject	Environmental Genomics and Metagenomics
Specific subject area	Metagenomics
Type of data	Tables, Images, Figures Raw, Analyzed, Filtered, Processed, and analyzed DNA sequencing data.
Data collection	One liter of water was sampled within a gutter along houses. Four hundred milliliters were successively filtered through two mixed cellulose ester filters of 5 µm, one of 1.2 µm, and three of 0.22 µm to recover the maximum of microorganisms. Total DNA was extracted using the DNeasy PowerWater kit. DNA library sequencing was performed on an Illumina HiSeq 4000 machine with paired-end 150 base sequencing reads.
Data source location	Water was sampled in the city of Pointe-Noire, Republic of Congo (precise coordinates: 4°48′43.1″S 11°52′27.8″E) under the supervision of the Direction Départementale des Soins et Services de Santé de Pointe Noire.
Data accessibility	The raw data files were deposited in the NCBI database under the study entitled “Metagenome from sample collected from water stream in Pointe-Noire city (Republic of Congo)”, BioProject No. PRJNA1021800. Supplementary tables (S1-S5) and refined and dereplicated (not assemblies paired) bins are available in the Zenodo data repository: https://zenodo.org/records/11278913 All the tools and command lines used in the present study are detailed and made public in a Github repository available at the following address: https://github.com/UMR-CNRS-7267/Metacongo_Paper

## 1 Value of the Data

• The dataset provides the first insights into the microbial diversity of gutter water from the city of Pointe-Noire, Republic of Congo.
• The discovery of pathogenic microorganisms could help local authorities anticipate epidemics' emergence.
• The dataset allows the identification of new metagenome-assembled genomes (MAGs) that are of interest to environmental microbiologists.
• The dataset serves as a valuable primary resource for assessing the presence of antibiotic-resistance genes, offering a useful tool for monitoring risks by public health authorities as already done in Kenya, Uganda, and Tanzania.

## 2 Background

**Fig. 1:**
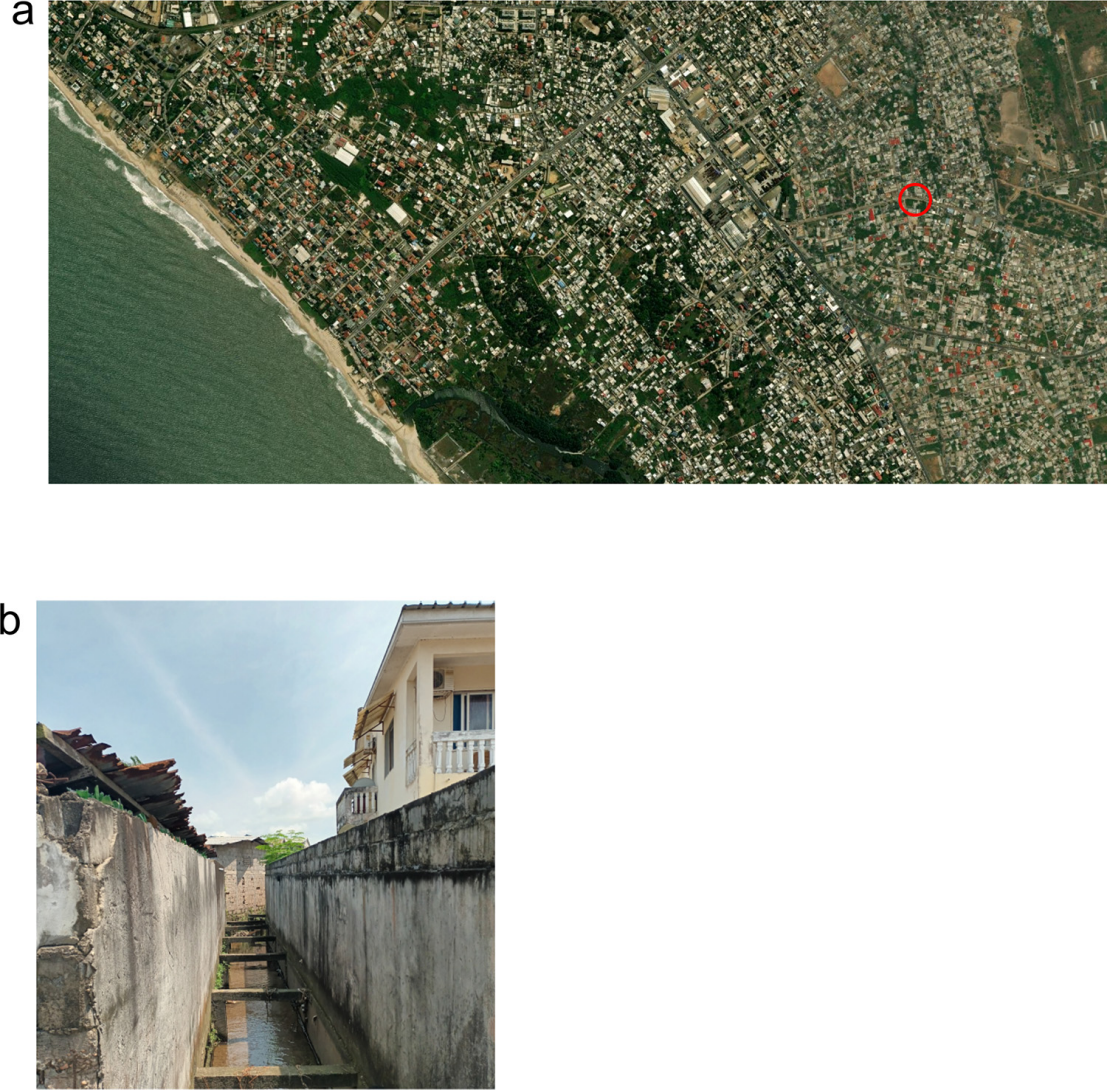
Geographical overview and location of the sampling site in Pointe-Noire. (a) The sampling was performed within the red circle area and the map was obtained from the website https://www.mapnall.com/en/. (b) photo of the gutter where water was sampled. (For interpretation of the references to color in this figure legend, the reader is referred to the web version of this article.)

The city prevents flood damage by digging gutters to drain excessive water. Sometimes people use these gutters to discharge numerous wastes including domestic wastewater. Thus, we selected one gutter point with a mix of water (rainwater and waste) to perform a preliminary study of the microbial composition useful for both environmental microbial ecology and public health authorities (Fig. 1). Results of such a project could convince public health authorities to extend the current analysis to different seasons or areas in the city of Pointe-Noire.

## 3 Data Description

The dataset is based on raw Illumina paired-end reads obtained through shotgun metagenomics sequencing of DNA isolated from gutter water collected in the city of Pointe-Noire. The raw data contain 84,886,827 paired-end reads of 150 bp (25,466 Mbases). The raw data used in this analysis and associated data analyses are available under NCBI BioProject No. PRJNA1021800.

**Fig. 2:**
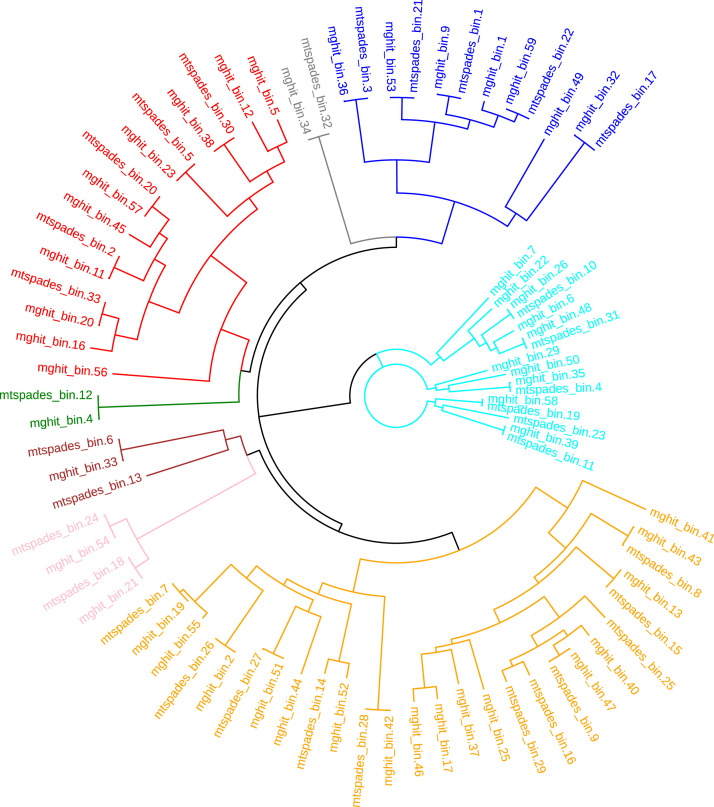
Phylogeny of identified bins (MAGs) using the FastTree software on the multi-alignment files generated by the pipeline. These MAGs belong to several phyla: Actinobacteriota (red); Bacteroidota (aqua); Bdellovibrionota (pink); Hydrogenedentota (gray); Myxococcota (brown); Planctomycetota (green); Proteobacteria (orange) and Verrucomicrobiota (blue). (For interpretation of the references to color in this figure legend, the reader is referred to the web version of this article.)

Regarding the taxonomic distribution, using the Kaiju profiler, we identified Bacteria, viruses, Archea, and Eukaryota. The list of the microbial taxonomy of identified organisms is provided in supplementary tables S1, S2, and S3. Unclassified reads were analysed with a second profiler (kraken 2) to extract the maximum information from the data. However, although some reads were assigned to bacteria, most of them remained unassigned (supplementary table S4). Furthermore, de novo assembly of the whole dataset allowed the identification of 81 metagenome-assembled genomes (Fig. 2, supplementary table S5) with an associated taxonomy described in Table 1.

**Table 1:** Taxonomic categories of the 81 MAGs identified.

BIN	DIVISION	PHYLUM	CLASS	ORDER	FAMILLY	GENUS	SPECIES	Others
mghit_bin.1	d__Bacteria	p__Verrucomicrobiota	c__Verrucomicrobiae	o__Verrucomicrobiales	f__Verrucomicrobiaceae	g__Prosthecobacter	s__	N/A
mghit_bin.11	d__Bacteria	p__Actinobacteriota	c__Actinomycetia	o__Actinomycetales	f__Microbacteriaceae	g__Schumannella	s__	N/A
mghit_bin.12	d__Bacteria	p__Actinobacteriota	c__Actinomycetia	o__Nanopelagicales	f__S36-B12	g__UBA6154	s__	N/A
mghit_bin.13	d__Bacteria	p__Proteobacteria	c__Gammaproteobacteria	o__Burkholderiales	f__Rhodocyclaceae	g__Paddy-1	s__	N/A
mghit_bin.16	d__Bacteria	p__Actinobacteriota	c__Actinomycetia	o__Actinomycetales	f__Microbacteriaceae	g__Rhodoluna	s__Rhodoluna	planktonica
mghit_bin.17	d__Bacteria	p__Proteobacteria	c__Gammaproteobacteria	o__Burkholderiales	f__Burkholderiaceae	g__SCGC-AAA027-K21	s__	N/A
mghit_bin.19	d__Bacteria	p__Proteobacteria	c__Alphaproteobacteria	o__Rhizobiales	f__Beijerinckiaceae	g__Alsobacter	s__	N/A
mghit_bin.2	d__Bacteria	p__Proteobacteria	c__Alphaproteobacteria	o__Rhodobacterales	f__Rhodobacteraceae	g__TH137	s__	N/A
mghit_bin.20	d__Bacteria	p__Actinobacteriota	c__Actinomycetia	o__Actinomycetales	f__Microbacteriaceae	g__Rhodoluna	s__	N/A
mghit_bin.21	d__Bacteria	p__Bdellovibrionota	c__Bacteriovoracia	o__Bacteriovoracales	f__Bacteriovoracaceae	g__UBA4096	s__	N/A
mghit_bin.22	d__Bacteria	p__Bacteroidota	c__Bacteroidia	o__Chitinophagales	f__Chitinophagaceae	g__RDXD01	s__	N/A
mghit_bin.23	d__Bacteria	p__Actinobacteriota	c__Actinomycetia	o__Nanopelagicales	f__Nanopelagicaceae	g__	s__	N/A
mghit_bin.25	d__Bacteria	p__Proteobacteria	c__Gammaproteobacteria	o__Burkholderiales	f__Burkholderiaceae	g__Polynucleobacter	s__	N/A
mghit_bin.26	d__Bacteria	p__Bacteroidota	c__Bacteroidia	o__Chitinophagales	f__Chitinophagaceae	g__SXYR01	s__	N/A
mghit_bin.29	d__Bacteria	p__Bacteroidota	c__Bacteroidia	o__AKYH767	f__Palsa-965	g__GCA-2737665	s__	N/A
mghit_bin.32	d__Bacteria	p__Verrucomicrobiota	c__Verrucomicrobiae	o__Opitutales	f__Opitutaceae	g__Opitutus	s__	N/A
mghit_bin.33	d__Bacteria	p__Myxococcota	c__UBA9042	o__UBA9042	f__UBA9042	g__	s__	N/A
mghit_bin.34	d__Bacteria	p__Hydrogenedentota	c__Hydrogenedentia	o__Hydrogenedentiales	f__SCBR16-9	g__	s__	N/A
mghit_bin.35	d__Bacteria	p__Bacteroidota	c__Bacteroidia	o__Flavobacteriales	f__Flavobacteriaceae	g__Flavobacterium	s__Flavobacterium	sp002422095
mghit_bin.36	d__Bacteria	p__Verrucomicrobiota	c__Verrucomicrobiae	o__Verrucomicrobiales	f__Akkermansiaceae	g__UBA1315	s__	N/A
mghit_bin.37	d__Bacteria	p__Proteobacteria	c__Gammaproteobacteria	o__Burkholderiales	f__Burkholderiaceae	g__	s__	N/A
mghit_bin.38	d__Bacteria	p__Actinobacteriota	c__Actinomycetia	o__Nanopelagicales	f__UBA5976	g__	s__	N/A
mghit_bin.39	d__Bacteria	p__Bacteroidota	c__Bacteroidia	o__NS11-12g	f__UBA955	g__	s__	N/A
mghit_bin.4	d__Bacteria	p__Planctomycetota	c__Planctomycetes	o__Isosphaerales	f__Isosphaeraceae	g__QWQI01	s__	N/A
mghit_bin.40	d__Bacteria	p__Proteobacteria	c__Gammaproteobacteria	o__Burkholderiales	f__Burkholderiaceae	g__Limnohabitans	s__	N/A
mghit_bin.41	d__Bacteria	p__Proteobacteria	c__Gammaproteobacteria	o__Methylococcales	f__Methylococcaceae	g__UBA6136	s__	N/A
mghit_bin.42	d__Bacteria	p__Proteobacteria	c__Alphaproteobacteria	o__Rickettsiales	f__Midichloriaceae	g__	s__	N/A
mghit_bin.43	d__Bacteria	p__Proteobacteria	c__Gammaproteobacteria	o__Burkholderiales	f__Methylophilaceae	g__Methylopumilus_A	s__	N/A
mghit_bin.44	d__Bacteria	p__Proteobacteria	c__Alphaproteobacteria	o__Acetobacterales	f__Acetobacteraceae	g__Ga0074136	s__	N/A
mghit_bin.45	d__Bacteria	p__Actinobacteriota	c__Actinomycetia	o__Actinomycetales	f__Microbacteriaceae	g__Aurantimicrobium	s__	N/A
mghit_bin.46	d__Bacteria	p__Proteobacteria	c__Gammaproteobacteria	o__Burkholderiales	f__Burkholderiaceae	g__SCGC-AAA027-K21	s__SCGC-AAA027-K21	sp003507735
mghit_bin.47	d__Bacteria	p__Proteobacteria	c__Gammaproteobacteria	o__Burkholderiales	f__Burkholderiaceae	g__Limnohabitans	s__	N/A
mghit_bin.48	d__Bacteria	p__Bacteroidota	c__Bacteroidia	o__Chitinophagales	f__Chitinophagaceae	g__JJ008	s__	N/A
mghit_bin.49	d__Bacteria	p__Verrucomicrobiota	c__Verrucomicrobiae	o__Pedosphaerales	f__UBA9464	g__SXXZ01	s__	N/A
mghit_bin.5	d__Bacteria	p__Actinobacteriota	c__Actinomycetia	o__Nanopelagicales	f__S36-B12	g__UBA10649	s__	N/A
mghit_bin.50	d__Bacteria	p__Bacteroidota	c__Bacteroidia	o__Flavobacteriales	f__UA16	g__UBA4660	s__	N/A
mghit_bin.51	d__Bacteria	p__Proteobacteria	c__Alphaproteobacteria	o__Reyranellales	f__Reyranellaceae	g__Reyranella	s__	N/A
mghit_bin.52	d__Bacteria	p__Proteobacteria	c__Alphaproteobacteria	o__Sphingomonadales	f__Sphingomonadaceae	g__Novosphingobium	s__	N/A
mghit_bin.53	d__Bacteria	p__Verrucomicrobiota	c__Verrucomicrobiae	o__Verrucomicrobiales	f__Verrucomicrobiaceae	g__Prosthecobacter	s__	N/A
mghit_bin.54	d__Bacteria	p__Bdellovibrionota	c__Bacteriovoracia	o__Bacteriovoracales	f__Bacteriovoracaceae	g__UBA4096	s__	N/A
mghit_bin.55	d__Bacteria	p__Proteobacteria	c__Alphaproteobacteria	o__Rhizobiales	f__Beijerinckiaceae	g__Alsobacter	s__	N/A
mghit_bin.56	d__Bacteria	p__Actinobacteriota	c__Acidimicrobiia	o__Acidimicrobiales	f__Ilumatobacteraceae	g__UBA668	s__	N/A
mghit_bin.57	d__Bacteria	p__Actinobacteriota	c__Actinomycetia	o__Actinomycetales	f__Microbacteriaceae	g__Aurantimicrobium	s__Aurantimicrobium	minutum
mghit_bin.58	d__Bacteria	p__Bacteroidota	c__Bacteroidia	o__Sphingobacteriales	f__	g__	s__	N/A
mghit_bin.59	d__Bacteria	p__Verrucomicrobiota	c__Verrucomicrobiae	o__Verrucomicrobiales	f__Verrucomicrobiaceae	g__Prosthecobacter	s__	N/A
mghit_bin.6	d__Bacteria	p__Bacteroidota	c__Bacteroidia	o__Chitinophagales	f__Chitinophagaceae	g__UBA3961	s__	N/A
mghit_bin.7	d__Bacteria	p__Bacteroidota	c__Bacteroidia	o__Chitinophagales	f__Saprospiraceae	g__M3007	s__	N/A
mghit_bin.9	d__Bacteria	p__Verrucomicrobiota	c__Verrucomicrobiae	o__Verrucomicrobiales	f__Verrucomicrobiaceae	g__Prosthecobacter	s__	N/A
mtspades_bin.1	d__Bacteria	p__Verrucomicrobiota	c__Verrucomicrobiae	o__Verrucomicrobiales	f__Verrucomicrobiaceae	g__Prosthecobacter	s__	N/A
mtspades_bin.10	d__Bacteria	p__Bacteroidota	c__Bacteroidia	o__Chitinophagales	f__Chitinophagaceae	g__SXYR01	s__	N/A
mtspades_bin.11	d__Bacteria	p__Bacteroidota	c__Bacteroidia	o__NS11-12g	f__UBA955	g__	s__	N/A
mtspades_bin.12	d__Bacteria	p__Planctomycetota	c__Planctomycetes	o__Isosphaerales	f__Isosphaeraceae	g__QWQI01	s__	N/A
mtspades_bin.13	d__Bacteria	p__Myxococcota	c__Myxococcia	o__Myxococcales	f__Myxococcaceae	g__Archangium_A	s__	N/A
mtspades_bin.14	d__Bacteria	p__Proteobacteria	c__Alphaproteobacteria	o__Sphingomonadales	f__Sphingomonadaceae	g__Novosphingobium	s__	N/A
mtspades_bin.15	d__Bacteria	p__Proteobacteria	c__Gammaproteobacteria	o__Burkholderiales	f__Rhodocyclaceae	g__Paddy-1	s__	N/A
mtspades_bin.16	d__Bacteria	p__Proteobacteria	c__Gammaproteobacteria	o__Burkholderiales	f__Burkholderiaceae	g__Limnohabitans	s__	N/A
mtspades_bin.17	d__Bacteria	p__Verrucomicrobiota	c__Verrucomicrobiae	o__Opitutales	f__Opitutaceae	g__Opitutus	s__	N/A
mtspades_bin.18	d__Bacteria	p__Bdellovibrionota	c__Bacteriovoracia	o__Bacteriovoracales	f__Bacteriovoracaceae	g__UBA4096	s__	N/A
mtspades_bin.19	d__Bacteria	p__Bacteroidota	c__Bacteroidia	o__Sphingobacteriales	f__	g__	s__	N/A
mtspades_bin.2	d__Bacteria	p__Actinobacteriota	c__Actinomycetia	o__Actinomycetales	f__Microbacteriaceae	g__Schumannella	s__	N/A
mtspades_bin.20	d__Bacteria	p__Actinobacteriota	c__Actinomycetia	o__Actinomycetales	f__Microbacteriaceae	g__Aurantimicrobium	s__Aurantimicrobium	minutum
mtspades_bin.21	d__Bacteria	p__Verrucomicrobiota	c__Verrucomicrobiae	o__Verrucomicrobiales	f__Verrucomicrobiaceae	g__Prosthecobacter	s__	N/A
mtspades_bin.22	d__Bacteria	p__Verrucomicrobiota	c__Verrucomicrobiae	o__Verrucomicrobiales	f__Verrucomicrobiaceae	g__Prosthecobacter	s__	N/A
mtspades_bin.23	d__Bacteria	p__Bacteroidota	c__Bacteroidia	o__Cytophagales	f__Spirosomaceae	g__UBA6715	s__	N/A
mtspades_bin.24	d__Bacteria	p__Bdellovibrionota	c__Bacteriovoracia	o__Bacteriovoracales	f__Bacteriovoracaceae	g__UBA4096	s__	N/A
mtspades_bin.25	d__Bacteria	p__Proteobacteria	c__Gammaproteobacteria	o__Burkholderiales	f__Burkholderiaceae	g__JOSHI-001	s__	N/A
mtspades_bin.26	d__Bacteria	p__Proteobacteria	c__Alphaproteobacteria	o__Rhodobacterales	f__Rhodobacteraceae	g__TH137	s__	N/A
mtspades_bin.27	d__Bacteria	p__Proteobacteria	c__Alphaproteobacteria	o__Reyranellales	f__Reyranellaceae	g__Reyranella	s__	N/A
mtspades_bin.28	d__Bacteria	p__Proteobacteria	c__Alphaproteobacteria	o__Rickettsiales	f__Midichloriaceae	g__	s__	N/A
mtspades_bin.29	d__Bacteria	p__Proteobacteria	c__Gammaproteobacteria	o__Burkholderiales	f__Burkholderiaceae	g__Limnohabitans	s__	N/A
mtspades_bin.3	d__Bacteria	p__Verrucomicrobiota	c__Verrucomicrobiae	o__Verrucomicrobiales	f__Akkermansiaceae	g__UBA1315	s__	N/A
mtspades_bin.30	d__Bacteria	p__Actinobacteriota	c__Actinomycetia	o__Nanopelagicales	f__UBA5976	g__	s__	N/A
mtspades_bin.31	d__Bacteria	p__Bacteroidota	c__Bacteroidia	o__Chitinophagales	f__Chitinophagaceae	g__JJ008	s__	N/A
mtspades_bin.32	d__Bacteria	p__Hydrogenedentota	c__Hydrogenedentia	o__Hydrogenedentiales	f__	g__	s__	N/A
mtspades_bin.33	d__Bacteria	p__Actinobacteriota	c__Actinomycetia	o__Actinomycetales	f__Microbacteriaceae	g__Rhodoluna	s__	N/A
mtspades_bin.4	d__Bacteria	p__Bacteroidota	c__Bacteroidia	o__Flavobacteriales	f__Flavobacteriaceae	g__Flavobacterium	s__Flavobacterium	sp002422095
mtspades_bin.5	d__Bacteria	p__Actinobacteriota	c__Actinomycetia	o__Nanopelagicales	f__Nanopelagicaceae	g__	s__	N/A
mtspades_bin.6	d__Bacteria	p__Myxococcota	c__UBA9042	o__UBA9042	f__UBA9042	g__	s__	N/A
mtspades_bin.7	d__Bacteria	p__Proteobacteria	c__Alphaproteobacteria	o__Rhizobiales	f__Beijerinckiaceae	g__Alsobacter	s__	N/A
mtspades_bin.8	d__Bacteria	p__Proteobacteria	c__Gammaproteobacteria	o__Burkholderiales	f__Methylophilaceae	g__Methylopumilus_A	s__	N/A
mtspades_bin.9	d__Bacteria	p__Proteobacteria	c__Gammaproteobacteria	o__Burkholderiales	f__Burkholderiaceae	g__Limnohabitans	s__	N/A

Table 1. List and taxonomy of MAGs.

Our study also included a screening of antibiotic-resistance genes in the whole assembly, with 27 antibiotic-resistance genes identified and listed in Table 2.

**Table 2:** Accession number, gene functions and antibiotic resistance.

Table 2. Identification and characterization of antibiotic-resistance genes.

## 4 Experimental Design, Materials and Methods

### 4.1 Sample collection

For this preliminary study, one liter of water was sampled once on September 19^th^, 2019 within a gutter along houses in the city of Pointe-Noire (latitude and longitude 4°48′43.1″S 11°52′27.8″E) in the Republic of Congo. Water was transported in a bottle with an iced pack and stored at 4 °C for six days until DNA extraction.

### 4.2 DNA isolation, library preparation, and shotgun sequencing

Four hundred milliliter of water were successively filtered through two mixed cellulose esters filters of 5 µm, one of 1.2 µm, and three of 0.22 µm (MCE membrane; 47 mm; MF-Millipore). DNA was extracted and pooled from the six filters using the DNeasy PowerWater kit (Qiagen) according to the manufacturer's instructions. Library preparation and sequencing were performed by GENEWIZ, from Azenta Life Sciences company^Ⓡ^. DNA library sequencing was performed on an Illumina HiSeq 4000 machine in paired-end mode producing reads of 150 base pairs length. The raw data contain 2 × 84,886,827 paired-end reads of 150 bp (25,466 Mbases).

### 4.3 Data preprocessing and cleaning

The quality of sequencing data was assessed and visualized using the fastqc tool v0.11.8 (http://www.bioinformatics.babraham.ac.uk/projects/fastqc/). To filter out adapters, undetermined bases, and poor-quality sequences, reads were subjected to a cleaning process using Fastp tool v0.23.2 [1]. Only sequences with a length greater than or equal to 50 bp were kept for analysis. Possible human sequences were filtered by a mapping approach using bowtie2 v2.3.4.3 [2] on the GRCh38 version of the human genome index available here (https://benlangmead.github.io/aws-indexes/bowtie). To extract unmapped reads, samtools v1.9 tool was used [3].

### 4.4 Metagenomics profiling

Two profilers were used Kaiju [4] and kraken2 [5]. The latter was used on reads that were not classified by the first profiler in order to minimize false positives. We used three available databases for Kaiju, nr_euk (version 2022-03-10) a database like NR (Non-Redundant Protein database but includes fungi and microbial eukaryotes), rvdb, (version 2022-04-07) which is Reference Viral Database [6] and finally a plasmids database (version 2022-04-10). All these databases are available on Kaiju homepage. All the classifications were merged into one file at the end of the analyses.

For these classifications reads that were not classified in the first database, were used in the second, and so on. Seqtk v 1.3-r106 (https://github.com/lh3/seqtk) tool extracted non-assigned reads in each step and prepared the input to the next one.

All reads unclassified by Kaiju were employed as inputs for profiling with Kraken2, a nucleic-based classifier utilizing a k-mer-based similarity approach. The database used in conjunction with Kraken2 is the PlusPF database, encompassing the standard Kraken2 database (RefSeq archaea, bacteria, viral, plasmid, human, UniVec_Core) along with Ref-Seq protozoa and fungi. The indexes of this database and others are freely available at https://benlangmead.github.io/aws-indexes/k2.

### 4.5 Metagenome assembled genomes (MAGs) reconstruction, taxonomic assignment, and functional annotation

Filtered and decontaminated reads were assembled by MEGAHIT [7] using kmer values ranging from 21 to 127 with a step of 2. Contigs with lengths inferior to 200 base pairs were discarded. In the same way and with the same kmer parameters, the metaSPAdes assembler [8] was used to generate the second assembly. The two binners used on each assembly (the so-called megahit assembly and the metaSPAdes assembly) are MaxBin2 [9] and Metabat2 [10]. They are widely used in binning and routinely are integrated into many metagenomic data analysis pipelines. They work in much the same way but with different sensitivities. The only notable difference between the two tools is the minimum length of contigs accepted by the binner. For metabat2, all contigs with a length < 1500 base pairs are filtered and not binned.

We then used two assemblers and two binners, resulting in four binned assemblies. The goal was to refine these assemblies to extract the maximum information from our dataset, particularly given the limitation of having only one sample. Subsequently, four modules from the MetaWRAP v1.1.2 [11] pipeline were applied to the bins. The bin refinement stage was performed using a MetaWRAP module (bin refinement module), where dereplication is performed. This module combines bins to create hybrid bins after evaluating the quality of each bin using CheckM. It then removes duplicate contigs appearing in multiple bins to ultimately identify the best version of each bin. For taxonomic assignment, we used also gtdbtk v 1.4.1 [[12], [13], [14], [15], [16]], using the whole pipeline to place contigs/bins in the GTDB reference tree. Contigs of each bin were annotated using prokka annotation pipeline v1.12 [17].

### 4.6 Screening of antibiotic resistance genes

MMseqs2 (release 14-7e284) [18] was employed to cluster the two assemblies (mega-hit and metaspades). Subsequently, the clustered assembly was used for the search for antimicrobial resistance genes. For this purpose, we used ABRicate (release 12), which can be found at https://github.com/tseemann/abricate.

## Limitations

The sampled water was stored at 4 °C for 6 days. Thus, organisms with a greater tolerance to cold temperatures may have taken advantage of the nutrients in the water and less competition to bloom which affects the relative abundance of bins.

## Ethics Statement

The authors have read and follow the ethical requirements for publication in Data in Brief and confirming that the current work does not involve human subjects, animal experiments, or any data collected from social media platforms.

## CRediT Author Statement

**Ascel Samba-Louaka**: Conceptualization, Supervision, Project administration, Resources, Funding acquisition, Writing - original draft; **Bouziane Moumen**: Formal analysis, Writing - original draft; **Céline Samba-Louaka**: Methodology, Ressources, review & editing; Anicet Magloire Boumba and Hervé Sabin Ngoma: review & editing; Aubierge Kimpamboudi: Project administration, Formal analysis, Writing – review & editing.

## Data Availability

Metagenome from sample collected from water stream in Pointe-Noire city (Republic of Congo) (Original data) (NCBI).

## Appendix Supplementary materials

Image, application 1

Image, application 2

Image, application 3

Image, application 4

Image, application 5

Image, application 6

## References

[1] Chen S. (2023). Ultrafast one-pass FASTQ data preprocessing, quality control, and deduplication using fastp. iMeta.

[2] Langmead B., Salzberg S.L. (2012). Fast gapped-read alignment with Bowtie 2. Nat. Methods.

[3] Danecek P., Bonfield J.K., Liddle J., Marshall J., Ohan V., Pollard M.O., Whitwham A., Keane T., McCarthy S.A., Davies R.M., Li H. (2021). Twelve years of SAMtools and BCFtools. Gigascience.

[4] Menzel P., Ng K.L., Krogh A. (2016). Fast and sensitive taxonomic classification for metagenomics with Kaiju. Nat. Commun..

[5] Wood D.E., Lu J., Langmead B. (2019). Improved metagenomic analysis with Kraken 2. Genome Biol..

[6] Goodacre N., Aljanahi A., Nandakumar S., Mikailov M., Khan A.S. (2018). A reference viral database (RVDB) to enhance bioinformatics analysis of high-throughput sequencing for novel virus detection. mSphere.

[7] Li D., Liu C.-M., Luo R., Sadakane K., Lam T.-W. (2015). MEGAHIT: an ultra-fast single-node solution for large and complex metagenomics assembly via succinct *de Bruijn* graph. Bioinformatics.

[8] Nurk S., Meleshko D., Korobeynikov A., Pevzner P.A. (2017). metaSPAdes: a new versatile metagenomic assembler. Genome Res..

[9] Wu Y.-W., Simmons B.A., Singer S.W. (2016). MaxBin 2.0: an automated binning algorithm to recover genomes from multiple metagenomic datasets. Bioinformatics.

[10] Kang D.D., Li F., Kirton E., Thomas A., Egan R., An H., Wang Z. (2019). MetaBAT 2: an adaptive binning algorithm for robust and efficient genome reconstruction from metagenome assemblies. PeerJ.

[11] Uritskiy G.V., DiRuggiero J., Taylor J. (2018). MetaWRAP—A flexible pipeline for genome-resolved metagenomic data analysis. Microbiome.

[12] Chaumeil P.-A., Mussig A.J., Hugenholtz P., Parks D.H. (2022). GTDB-Tk v2: memory friendly classification with the genome taxonomy database. Bioinformatics.

[13] Parks D.H., Chuvochina M., Rinke C., Mussig A.J., Chaumeil P.-A., Hugenholtz P. (2022). GTDB: an ongoing census of bacterial and archaeal diversity through a phylogenetically consistent, rank normalized and complete genome-based taxonomy. Nucleic Acids Res..

[14] Price M.N., Dehal P.S., Arkin A.P. (2010). FastTree 2 – approximately maximum-likelihood trees for large alignments. PLoS ONE.

[15] Eddy S.R. (2011). Accelerated profile HMM searches. PLoS Comput. Biol..

[16] Ondov B.D., Treangen T.J., Melsted P., Mallonee A.B., Bergman N.H., Koren S., Phillippy A.M. (2016). Mash: fast genome and metagenome distance estimation using MinHash. Genome Biol..

[17] Seemann T. (2014). Prokka: rapid prokaryotic genome annotation. Bioinformatics.

[18] Steinegger M., Söding J. (2017). MMseqs2 enables sensitive protein sequence searching for the analysis of massive data sets. Nat. Biotechnol..

